# Supplementary material for: Human cooperation in changing groups in a large-scale public goods game
Source: Nat Commun. 2022 Oct 27;13:6399. doi: 10.1038/s41467-022-34160-5 (PMC9613774; doi:10.1038/s41467-022-34160-5)
Supplement: Supplementary file 1 — Supplementary Information [file 41467_2022_34160_MOESM1_ESM.pdf]

Supplementary Information for:

Human cooperation in changing groups in a large-scale public goods game

Table of contents

Supplementary Methods

1. More details on public good provision in Ikariam

2. Thresholds and step-returns

Supplementary Notes

1. Relationship between total number of newcomers and group-average contributions

2. Robustness analyses

3. Analyses for the incumbent-newcomer difference in contribution behavior

Supplementary Methods

1. More details on public good provision in Ikariam

In Table S1, we show an example of what group members see about the contributions to the sawmill. In this example, the current public good level is 24, a total contribution of 1855942 units of wood is necessary to move from public good level 24 to 25, of which 700909 have been contributed so far (column 1 in Table S1). The total prior contributions that have been made to get the sawmill from level 1 to 24 are also displayed (column 6 in Table S1). We can see there that 4 of the 6 group members have contributed to the public good so far. Members are known to each other via self-chosen nicknames and names of their towns (columns 2-3 in Table S1). If persons look at the contributions of other group members, they can also see each other's (town hall) level, indicating how far they have progressed in the game (column 4 in Table S1).

The production rate of the public good is the same for each group member, but it is up to each member whether to collect what has been produced for him/her. Each group member has simulated citizens at his/her disposal that can be assigned as workers to gather the resources produced by the public goods or to do other tasks. For example, if a public good is at level 1 and therefore produces 30 units of wood per hour for each member, some members may still choose not to obtain their 30 units of wood per hour by not assigning their simulated citizens as workers to gather the wood. A person may choose to do so, for example, because the person wants to use his/her simulated citizens for other tasks, e.g., as soldiers to fight other persons. If persons look at the contributions of other group members, they can see how many workers each member has assigned to the public good (column 5 in Table S1). Note that the number of workers a member has assigned to the public good does not affect how much other members can collect from the public good, i.e., the good is non-rival.

In total, persons thus see their group members' nicknames, town nicknames, contributions to the public good, individual level, and usage of the public good. This works exactly the same for the sawmill and the island-specific public good (wine, marble, crystal glass, or sulfur). More information on the group structure can be found in Figure S1.

Table S1. Example table of public good provision in Ikariam

**Saw mill**	**Other players on this island**				
**Level:**	**Player**	**Town**	**Level**	**Workers**	**Donated**
24	Atlas-2812	Colony 42	Level 28	313 workers	0
**Required for next level:**	Brochis	Delta	Level 8	100 workers	50,000
1,855,942	Esprit	Ophelia	Level 30	655 workers	100,909
**Available:**	jessZeta	Zeta 1	Level 26	683 workers	551,484
700,909		Zeta 2	Level 26	696 workers	
Donations:		Zeta 3	Level 20	340 workers	
	Maspero	Judah	Level 16	0 workers	450,000
Donate for expansion		Sumeria	Level 29	696 workers	
	pseudonym	Polis	Level 1	0 workers	0

Column 1 shows the level of one of the public goods (the sawmill, level 24), the threshold to reach the next level of the public good (1855942 units of wood), and the resources already contributed to the public good (700909 units of wood). Columns 2-5 shows player nicknames, towns that are on this island, level of towns, and workers assigned to make use of the public good. Column 6 shows the total of contributions to the public good of all players during their time on the island (e.g. player ‘Esprit’ contributed 100909, whereas ‘pseudonym’ contributed 0).

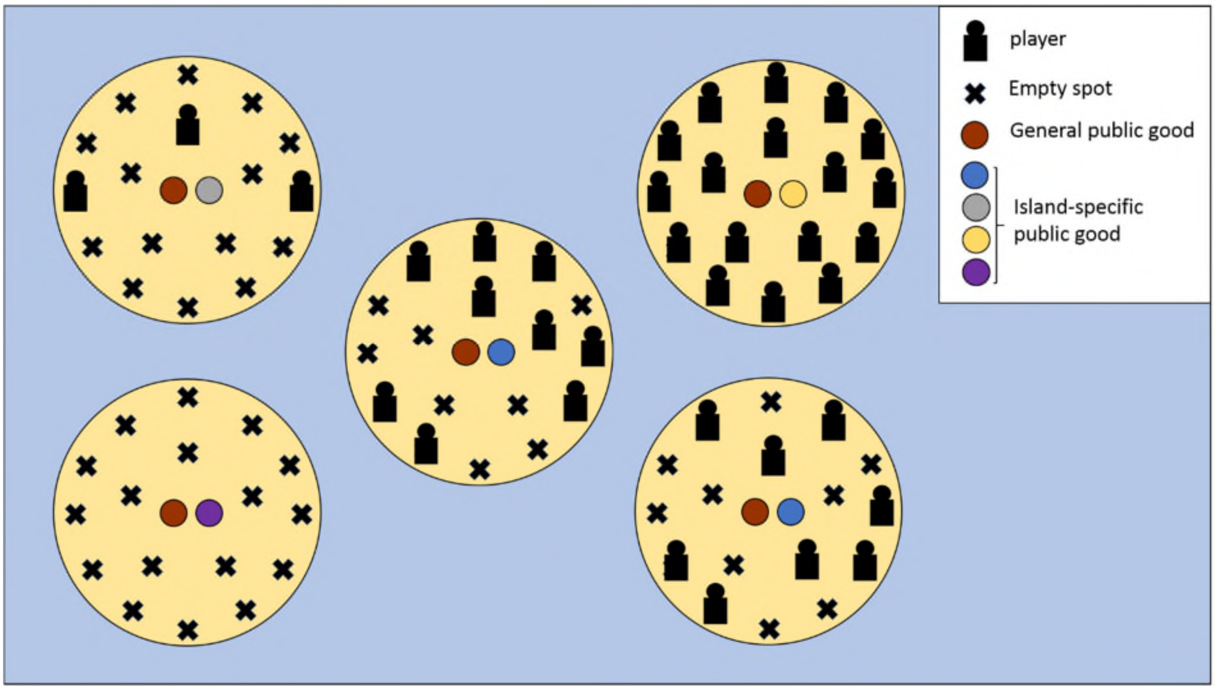

Figure S1. Example groups in Ikariam and their public goods. Public good provision occurs on islands in Ikariam. Each island contains two public goods. The first is the same on every island, namely a sawmill that provides wood (indicated with brown circles). The second public good provides an island-specific resource, namely crystal glass, marble, sulfur, or wine (indicated with blue, grey, yellow, and purple circles respectively). Up to 17 players can inhabit an island. Players have certain information available about other islands which they can use to decide which islands to inhabit next when building a new town. They know which players and how many players inhabit an island, which island-specific public good is produced on the island, the level of the public goods on the island, and how far the island is from the island(s) they currently inhabit. The players cannot see how much players are currently contributing to the public good on islands that they do not inhabit themselves, although the public good level may provide some indication of this. Players can also choose to build a new town on an island which they already inhabit.

2. Thresholds and step-returns

There are 50 thresholds (public good levels) in Ikariam that can be surpassed in succession. Each succeeding threshold requires more contributions than the previous threshold. For example, while increasing the public good from level 1 (return of 30 units of wood per hour) to level 2 (return of 38 units of wood per hour) requires only 394 contributed units of wood, further increasing to level 3 (return of 50 units of wood per hour) requires 992 contributed units of wood. Although increasing increments in thresholds are not common in public good games, some studies do incorporate them[1,2].

One important determinant of contributions to public goods is the value of the public good relative to the costs of producing it. For continuous public good games, this tradeoff between value and costs has been formalized in the multiplication factor and the marginal per capita return (MPCR). The parallel concept in threshold public good games is the step return, which is the total group payoff from the public good divided by the total contribution threshold[3].

In Ikariam, the total group payoff is mostly a function of time (and group size) because increasing the public good level increases the hourly production of resources. Since the multiplication factor and MPCR are about the tradeoff between value and costs, we denote the step return in Ikariam as the inverse of the number of days until the increase in total group payoffs from levelling up the public good matches the total contribution threshold. In Table S2, we provide the step returns for a medium-sized group (8 members) associated with each public good level. A step return of 0.2 would mean that it takes a medium-sized group 5 days until the increase in the total group payoffs from levelling up the public good breaks even with the contributions that were required to surpass the threshold.

Table S2. Thresholds and step-returns of public good provision

Public good level	Threshold	Step Return
1	-	-
2	394	4.678172589
3	992	2.787096774
4	1732	1.862355658
5	2788	1.322238164
6	3783	0.974464711
7	5632	0.736363636
8	8139	0.566162919
9	10452	0.44087256
10	13298	0.346518273
11	18478	0.274315402
12	23213	0.218360401
13	29038	0.174557476
14	39494	0.140011141
15	49107	0.112603091
16	66010	0.090749886
17	81766	0.073262725
18	101146	0.059225278
19	134598	0.04792939
20	154304	0.038822066
21	205012	0.031467426
22	270839	0.025520697
23	311541	0.020707387
24	411229	0.016808153
25	506475	0.013647268
26	665201	0.011083567
27	767723	0.009003247
28	1007959	0.007314583
29	1240496	0.005943429
30	1526516	0.004829822
31	1995717	0.003925206
32	2311042	0.003190249
33	3020994	0.002593054
34	3935195	0.002107748
35	4572136	0.001713335
36	5624478	0.001392769
37	7325850	0.00113221
38	9011590	0.000920415
39	11085051	0.000748251
40	13635408	0.000608299
41	17704143	0.000494528
42	20630781	0.00040204
43	26786470	0.000326852
44	32948197	0.000265726
45	40527121	0.000216033
46	52472840	0.000175634
47	61315353	0.00014279
48	79388129	0.000116088
49	97648282	0.000094380
50	120108270	0.000076731

Supplementary Notes

1. Relationship between total number of newcomers and group-average contributions

Table S3 shows the relationship between the group-average contribution percentage over all 28 time periods and the total number of newcomers that entered during this time. We see that there is a negative relationship between the number of newcomers and the average contribution percentage.

Table S3. Group-average contribution across all time periods by total number of newcomers, average group size, and average period

Total number of newcomers	-0.45***
	(0.03)
Average group size	-0.03
	(0.13)
Average period	0.94***
	(0.11)
Intercept	29.29***
	(2.11)
N observations	11348
R^2	0.06

* $p < 0.05$, ** $p < 0.01$, *** $p < 0.001$. Linear regression. Coefficients are marginal effects with standard errors in parentheses. Statistical significance is calculated using two-sided t-tests. All variables are entered without discretization.

2. Robustness analyses

We conduct several robustness analyses to examine whether the negative relationship between the number of newcomers and the contribution percentage also holds under different operationalizations of the number of newcomers and contribution behavior. First, we replace the dichotomous distinction between newcomers and incumbents with a continuous variable indicating what we call tenure. This variable counts how many prior time periods an individual has been present in the group. A longer average tenure in a group, when controlling for group size and period, indicates more stability in group composition. We show that a group's average tenure is positively related to its contribution percentage, suggesting that fewer group changes are related to higher contributions (Table S4). Second, we repeat the analyses for separate country-specific game servers (Table S5-S6) and the two public goods separately (Table S7-S9). Third, we repeat the analyses when taking the first-lag of the number of newcomers (Table S10). Fourth, we repeat the analyses when leaving out outliers with very high contribution percentages (Table S11). Fifth, we conduct analyses with crossed fixed effects that account simultaneously for between-group and between-individual confounders (Table S12-13). Finally, we conduct analyses in which we control for the public good level (Table S14). The negative relationship between the number of newcomers and contributions to the public goods is robust to all these different specifications.

Table S4. OLS regression model of the average contribution percentage by group tenure

Average tenure	3.84***
	(0.18)
Average group size	-3.07***
	(0.11)
Average snapshot	1.12***
	(0.10)
Intercept	15.72***
	(2.15)
N observations	11348
R^2	0.08

Note:* $p < 0.05$, ** $p < 0.01$, *** $p < 0.001$. Linear regression. Coefficients are marginal effects with standard errors in parentheses. Statistical significance is calculated using two-sided t-tests. We see that the average group tenure is positively related to the contribution percentage (B = 3.84, SE = 0.18, $p < .001$), indicating that more stability in group composition is associated with higher contribution percentages. All variables are entered without discretization.

Table S5. Contribution percentage by the number of newcomers per period, separated by country

	(1) Germany	(2) England	(3) France	(4) Greece	(5) Turkey
Number of newcomers	-2.73***	-3.87***	-2.65***	-2.70***	-2.22***
	(0.09)	(0.12)	(0.09)	(0.10)	(0.06)
Group size	-1.04***	0.02	-0.75***	0.24***	-0.08*
	(0.06)	(0.09)	(0.06)	(0.06)	(0.04)
Period	-0.37***	-0.46***	-0.23***	-0.18***	-0.17***
	(0.03)	(0.04)	(0.03)	(0.03)	(0.02)
Intercept	32.97***	41.30***	35.30***	37.61***	27.04***
	(0.16)	(0.23)	(0.16)	(0.18)	(0.11)
N observations	35728	29801	37256	37176	59569
R^2 (overall)	0.06	0.03	0.06	0.02	0.03
R^2 (within)	0.04	0.04	0.03	0.02	0.03

Note:* $p < 0.05$, ** $p < 0.01$, *** $p < 0.001$. Linear regression with group fixed effects to account for repeated measures within groups. Coefficients are marginal effects with standard errors in parentheses. Statistical significance is calculated using two-sided t-tests. The units of analysis are groups per period. We have data for five country-specific servers, namely: Germany, England, France, Greece, and Turkey. The negative relationship between the number of newcomers and the contribution percentage holds in all of the five servers (Germany, B = -2.73, SE = 0.09, $p < .001$; England, B = -3.87, SE = 0.12, $p < .001$; France, B = -2.65, SE = 0.09, $p < .001$; Greece, B = -2.70, SE = 0.10, $p < .001$; Turkey, B = -2.22, SE = 0.06, $p < .001$). All variables are entered without discretization.

Table S6. Contribution percentage by the total number of newcomers across all periods, separated by country

	(1) Germany	(2) England	(3) France	(4) Greece	(5) Turkey
Total number of newcomers	-0.39***	-0.40***	-0.49***	-0.62***	-0.32***
	(0.08)	(0.08)	(0.10)	(0.11)	(0.04)
Average group size	-0.35	0.33	-0.59	0.70*	0.15
	(0.32)	(0.33)	(0.35)	(0.34)	(0.21)
Average period	1.09***	0.92***	0.41	0.53*	1.93***
	(0.24)	(0.26)	(0.26)	(0.26)	(0.19)
Intercept	36.10***	41.64***	38.61***	38.45***	29.07
	(0.73)	(1.00)	(0.87)	(0.89)	(0.54)
N observations	2119	1798	2184	2063	3184
R^2	0.07	0.03	0.06	0.03	0.09

Note:* $p < 0.05$, ** $p < 0.01$, *** $p < 0.001$. Linear regression. Coefficients are marginal effects with standard errors in parentheses. Statistical significance is calculated using two-sided t-tests. The units of analysis are groups. We have data for five country-specific servers, namely: Germany, England, France, Greece, and Turkey. The negative relationship between the number of newcomers and the contribution percentage holds in all of the five servers (Germany, B = -0.39, SE = 0.08, $p < .001$; England, B = -0.40, SE = 0.08, $p < .001$; France, B = -0.49, SE = 0.10, $p < .001$; Greece, B = -0.62, SE = 0.11, $p < .001$; Turkey, B = -0.32, SE = 0.04, $p < .001$). All variables are entered without discretization.

Table S7. Contribution percentage by the number of newcomers per period, separated by public good

	(1) sawmill	(2) island-specific good
Number of newcomers	-1.48***	-1.39***
	(0.02)	(0.02)
Group size	-0.15***	-0.05***
	(0.02)	(0.02)
Period	-0.39***	0.12***
	(0.01)	(0.01)
Intercept	18.20***	16.49***
	(0.05)	(0.04)
N observations	199582	199618
R^2 (overall)	0.02	0.04

Note:* $p < 0.05$, ** $p < 0.01$, *** $p < 0.001$. Linear regression with group fixed effects to account for repeated measures within groups. Coefficients of independent variables and intercept are marginal effects with standard errors in parentheses. Statistical significance is calculated using two-sided t-tests. The units of analysis are groups per period. Public good 1 is the sawmill, public good 2 is the island-specific public good (i.e., marble, crystal glass, sulfur, wine). The negative relationship between the contribution percentage and the number of newcomers holds for both public good types (sawmill, B = -1.48, SE = 0.02, $p < .001$; island-specific good, B = -1.39, SE = 0.02, $p < .001$). All variables are entered without discretization.

Table S8. Contribution percentage by the total number of newcomers across all periods, separated by public good

	(1) sawmill	(2) island-specific good
Total number of newcomers	-0.24***	-0.22***
	(0.02)	(0.02)
Average group size	0.15	-0.02
	(0.08)	(0.07)
Average period	0.12	0.82***
	(0.07)	(0.06)
Intercept	21.04***	8.26***
	(1.33)	(1.22)
N observations	11349	11348
R^2	0.03	0.06

Note:* $p < 0.05$, ** $p < 0.01$, *** $p < 0.001$. Linear regression. Coefficients of independent variables and intercept are marginal effects with standard errors in parentheses. Statistical significance is calculated using two-sided t-tests. The units of analysis are groups. Public good 1 is the sawmill, public good 2 is the island-specific public good (i.e., marble, crystal glass, sulfur, wine). The negative relationship between the contribution percentage and the number of newcomers holds for both public good types (sawmill, B = -0.24, SE = 0.02, $p < .001$; island-specific good, B = -0.22, SE = 0.02, $p < .001$). All variables are entered without discretization.

Table S9. Individual contribution percentage by newcomer status per public good

	(1) sawmill	(2) island-specific good
Newcomer	-10.55***	-8.45***
	(0.09)	(0.08)
Period	0.22***	0.55***
	(0.01)	(0.01)
Group size	-0.19***	-0.38***
	(0.01)	(0.01)
Intercept	16.62***	10.97***
	(0.17)	(0.16)
N observations	1576025	1581248
R^2 (overall)	0.01	0.01

Note:* $p < 0.05$, ** $p < 0.01$, *** $p < 0.001$. Linear regression with individual fixed effects to account for repeated measures within individuals. Coefficients are marginal effects with standard errors in parentheses. Statistical significance is calculated using two-sided t-tests. The units of analysis are individuals per period. All variables are entered without discretization.

Table S10. Average contribution by lagged number of newcomers, group size, and period

	Model 1	Model 2
Lagged number of newcomers	-1.69***	-2.66***
	(0.04)	(0.06)
Group size	-0.79***	-0.62***
	(0.03)	(0.03)
Period	-0.36***	-0.37***
	(0.01)	(0.01)
Lagged number of newcomers × group size		0.20***
		(0.01)
Lagged number of newcomers × period		-0.04***
		(0.01)
Intercept	34.21***	34.21***
	(0.07)	(0.07)
N observations	186992	186992
R^2 (overall)	0.05	0.05
Rho	0.63	0.63

Note:* $p < 0.05$, ** $p < 0.01$, *** $p < 0.001$. Linear regression with group fixed effects to account for repeated measures within groups. Coefficients of independent variables and intercept are marginal effects with standard errors in parentheses. Statistical significance is calculated using two-sided t-tests. Results include 10942 groups (fewer than in Table 1 because here we can only include groups that were present in at least two consecutive time periods due to taking the lag of the number of newcomers), with these groups existing on average for ~17 periods, giving a total number of observations of 186992 group-period combinations. We find a negative relationship between the lagged number of newcomers and the group-average contribution percentage (Model 1, B = -1.69, SE = 0.04, $p < .001$; Model 2, B = -2.66, SE = 0.06, $p < .001$). All variables are entered without discretization.

Because individuals can move resources between their groups, it can happen that they contribute more to the public good of a group than the total resources they had available in that group, i.e., individuals can end up with contribution percentages above 100 percent. Likewise, because we only have snapshots of an individual's available resources instead of a continuous-time overview of an individual's resources, it is possible that an individual had more (or fewer) resources available than we see at the snapshot, which can also lead to contribution percentages above 100 percent. This happens in 6.5% of our analyzed cases. In Table S11, we show that the negative relationship between the number of newcomers and the group-average contribution percentage is robust to adjusting for these outliers or excluding them.

Table S11. Contribution percentage by the number of newcomers, adjusting for outliers

	Model 1	Model 2	Model 3	Model 4
Number of newcomers	-2.32***	-1.89***	-1.43***	-1.97***
	(0.02)	(0.02)	(0.01)	(0.02)
Group size	-0.03*	-0.50***	-0.40***	0.23***
	(0.02)	(0.01)	(0.01)	(0.01)
Period	-0.16***	-0.21***	-0.13***	-0.06***
	(0.01)	(0.01)	(0.00)	(0.01)
Intercept	29.93***	25.11***	18.51***	25.82***
	(0.04)	(0.03)	(0.03)	(0.04)
N observations	199530	199530	193660	188463
R^2 (overall)	0.05	0.11	0.10	0.04
R^2 (within)	0.05	0.07	0.07	0.05

Note:* $p < 0.05$, ** $p < 0.01$, *** $p < 0.001$. Linear regression with group fixed effects to account for repeated measures within groups. Coefficients are marginal effects with standard errors in parentheses. Statistical significance is calculated using two-sided t-tests. In Model 1, we set group-average contribution percentages above 100% to 100%. In Model 2, we set individual contribution percentages above 100% to 100% before calculating the group-average contribution. In Model 3, we exclude individuals with contribution percentages above 100% when calculating the group-average contribution percentage. In Model 4, we exclude groups with average contribution percentages above 100%. We see that the negative relationship between the number of newcomers and the group-average contribution is strongly robust to adjusting for, or excluding, outliers (Model 1, B = -2.32, SE = 0.02, $p < .001$; Model 2, B = -1.89, SE = 0.02, $p < .001$; Model 3, B = -1.43, SE = 0.01, $p < .001$; Model 4, B = -1.97, SE = 0.02, $p < .001$). All variables are entered without discretization.

Table S12. Crossed fixed effects for contribution percentage by number of newcomers

Number of newcomers	-1.51***
	(0.02)
Group size	0.66***
	(0.02)
Period	1.42***
	(0.01)
Intercept	2.99***
	(0.28)
N observations	1492659
R^2	0.04

Note:* $p < 0.05$, ** $p < 0.01$, *** $p < 0.001$. Linear regression with crossed fixed effects to account for repeated measures within groups and individuals. Coefficients are marginal effects with standard errors in parentheses. Statistical significance is calculated using two-sided t-tests. There are 80075 singleton observations, which are excluded. We find a negative relationship between the number of newcomers and the contribution percentage (B = -1.51, SE = 0.02, $p < .001$). All variables are entered without discretization.

Table S13. Crossed fixed effects for contribution percentage by newcomer status

Newcomer	-13.42***
	(0.12)
Group size	0.35***
	(0.02)
Period	1.31***
	(0.01)
Intercept	7.50***
	(0.29)
N observations	1492659
R^2	0.05

Note:* $p < 0.05$, ** $p < 0.01$, *** $p < 0.001$. Linear regression with crossed fixed effects to account for repeated measures within groups and individuals. Coefficients are marginal effects with standard errors in parentheses. Statistical significance is calculated using two-sided t-tests. There are 80075 singleton observations, which are excluded. We find that newcomers have lower contribution percentages than incumbents (B = -13.42, SE = 0.12, $p < .001$). All variables are entered without discretization.

Table S14. Regression model of average contribution percentages with group fixed effects

	Model 1	Model 2
Number of newcomers	-2.72***	-1.35***
	(0.04)	(0.06)
Group size	-0.32***	-1.91***
	(0.03)	(0.03)
Period	-0.27***	-3.44***
	(0.01)	(0.03)
Public good level		5.67***
		(0.05)
Number of newcomers × Public good level		-0.14***
		(0.01)
Intercept	33.75***	33.75***
	(0.07)	(0.07)
R^2 (overall)	0.04	0.03
Rho	0.58	0.66

* $p < 0.05$, ** $p < 0.01$, *** $p < 0.001$. Linear regression with group fixed effects to account for repeated measures within groups. Coefficients of independent variables and intercept are marginal effects with standard errors in parentheses. Statistical significance is calculated using two-sided t-tests. Results include 11348 groups, with groups existing on average for 17-18 periods, giving a total number of observations of 199530 group-period combinations. All variables are entered without discretization.

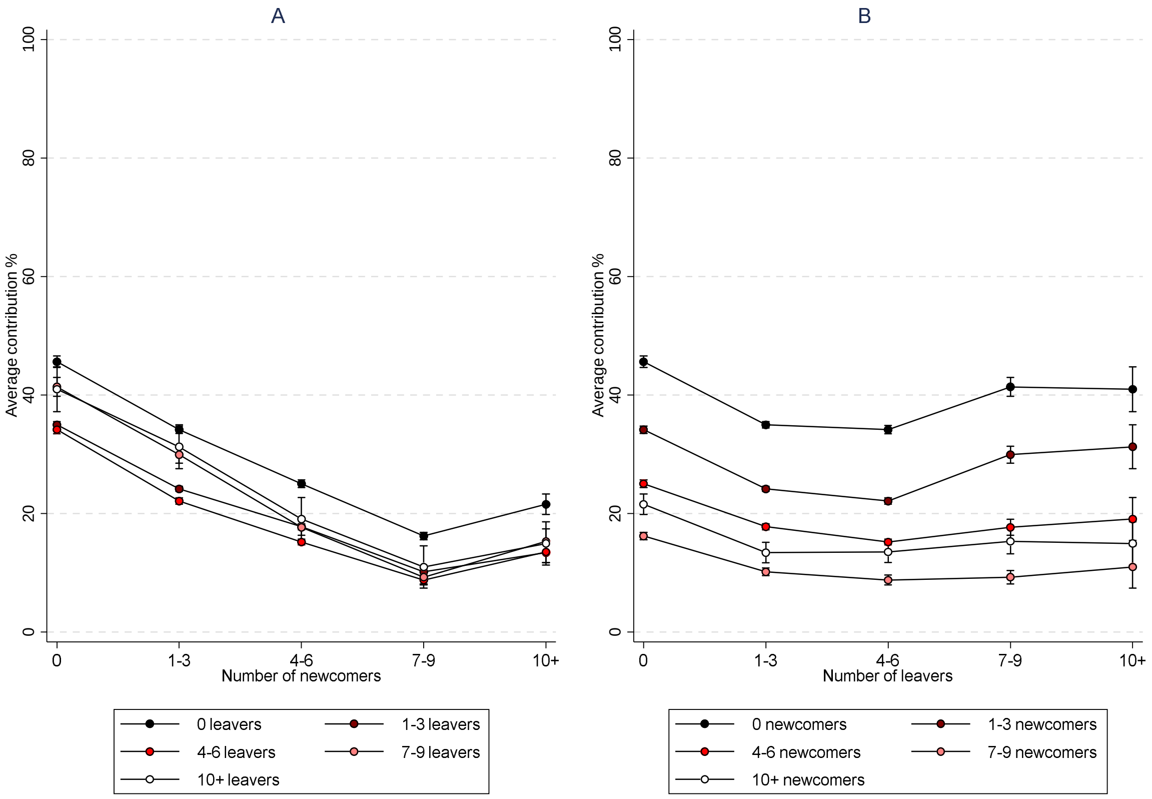

Figure S2. Contribution percentage by the number of newcomers and the number of leavers. We show the estimated margins of an OLS regression with the group's average contribution percentage as the dependent variable and the number of newcomers and the number of leavers (persons that left the group in the prior snapshot), group size, and snapshot as predictive factors (not including the interactions between the factors). The data are discretized in this figure and its underlying model (Table S15) for visualization purposes, the non-discretized analyses can be found in Table S16. Data are presented as mean values and we account for repeated observations within groups by estimating cluster-robust 95% confidence intervals. **(A)** We see that, controlling for the number of leavers, the number of newcomers has a negative relationship with the contribution percentage. **(B)** We see that, controlling for the number of newcomers, there is not a clear relationship between the number of leavers and the contribution percentage. The contribution decreases somewhat when moving from 0 to 1-3 leavers, but does not change much between 1-3 leavers and 10+ leavers. Results include 10942 groups, with groups existing on average for ~17 periods, giving a total number of observations of 187875 group-period combinations. The number of observations differs from analyses without the number of leavers, because we can only see if a group has leavers if the group exists in more than two periods, meaning that we have to leave out the few groups that exist for only one period (see also Table S15 and Table S16). Source data are provided as a Source Data file.

Table S15. Regression model underlying Figure S3.

1-3 newcomers	-8.73***
	(0.26)
4-6 newcomers	-15.50***
	(0.41)
7-9 newcomers	-23.76***
	(0.51)
10+ newcomers	-20.47***
	(0.96)
1-3 leavers	-7.05***
	(0.29)
4-6 leavers	-8.95***
	(0.35)
7-9 leavers	-7.30***
	(0.72)
10+ leavers	-7.92***
	(1.87)
Group sizes 6-10	-12.52***
	(0.59)
Group sizes 11-17	-11.26***
	(0.73)
Periods 6-10	-2.27***
	(0.44)
Periods 11-15	-4.23***
	(0.51)
Periods 16-20	-4.30***
	(0.55)
Periods 21-28	-6.07***
	(0.65)
Intercept	55.10***
	(0.84)
N observations	187875
R^2	0.07

Note:* $p < 0.05$, ** $p < 0.01$, *** $p < 0.001$. Linear regression. Coefficients are marginal effects with group cluster-robust standard errors in parentheses. Statistical significance is calculated using two-sided t-tests. Reference category for number of newcomers are groups with 0 newcomers, reference category for number of leavers are groups with 0 leavers, reference category for group size are groups with 1-5 members, reference category for time periods are groups at periods 1-5. Data were discretized for visualization of Figure S2, the non-discretized analyses are presented in Table S16.

Table S16. Regression model of average contribution percentages with group fixed effects

Number of newcomers	-2.76***
	(0.05)
Number of leavers	-1.10***
	(0.05)
Group size	-0.56***
	(0.03)
Period	-0.42***
	(0.01)
Intercept	34.16***
	(0.07)
R^2 (overall)	0.06
Rho	0.63

* $p < 0.05$, ** $p < 0.01$, *** $p < 0.001$. Linear regression with group fixed effects to account for repeated measures within groups. Coefficients of independent variables and intercept are marginal effects with standard errors in parentheses. Statistical significance is calculated using two-sided t-tests. Results include 10942 groups, with groups existing on average for ~17 periods, giving a total number of observations of 187875 group-period combinations. The number of observations differs from analyses without the number of leavers, because we can only see if a group has leavers if the group exists in more than two periods, meaning that we have to leave out the few groups that exist for only one period. Results show that the coefficient for the number of newcomers is 2-3 times the size of the coefficient for the number of leavers (B = -2.76, SE = 0.05, $p < .001$ and B = -1.10, SE = 0.05, $p < .001$ respectively). All variables are entered without discretization.

3. Analyses for the incumbent-newcomer difference in contribution behavior

In Table S17, we examine the relationship between the contribution percentages of individual players and their group members and how this depends on whether players are newcomers and time spent in the group. In Table S18, we examine if newcomers contribute higher percentages if they already know incumbents from their other groups. In Table S19, we examine the newcomer-incumbent contribution difference when selecting players who belong to only one group. In Table S20, we examine whether newcomers' contribution is related to the group-average contribution in their prior groups. In Table S21, we examine to what extent the difference in contribution percentage between newcomers and incumbents remains when adjusting for private good levels. A player's private good level on an island is captured by the player's town hall level on the island. Every increase in the town hall level increases the maximum number of citizens allowed in one's town. Each citizen works to produce resources and pays taxes to give gold (the latter of which can be used to buy yet more resources). Hence, a higher level town hall means more resources. In this sense, the town hall level is akin to the endowment in regular public good games. Comparing newcomers and incumbents of similar private good levels (town hall levels) is not trivial because incumbents typically have higher private good levels, as is shown in Figure S3. Especially at the high end of the private good levels, there are almost no newcomers (the converse is less problematic: there are enough incumbents with low private good levels). We see that town hall levels above 10 are achieved by only 1% of the newcomers, making it difficult to find enough newcomers above this level to compare with incumbents of the same town hall level. Therefore, when adding private good level as a control variable in Table S21, we restrict the analyses to town hall levels between 1 and 10, which captures 99% of the newcomers and 50% of the incumbents. In Table S22, we examine to what extent the difference in contribution percentage between newcomers and incumbents remains when adjusting for public good levels. Because the public good level does not differ between newcomers and incumbents, we do not have to restrict this analysis to a certain range of public good levels.

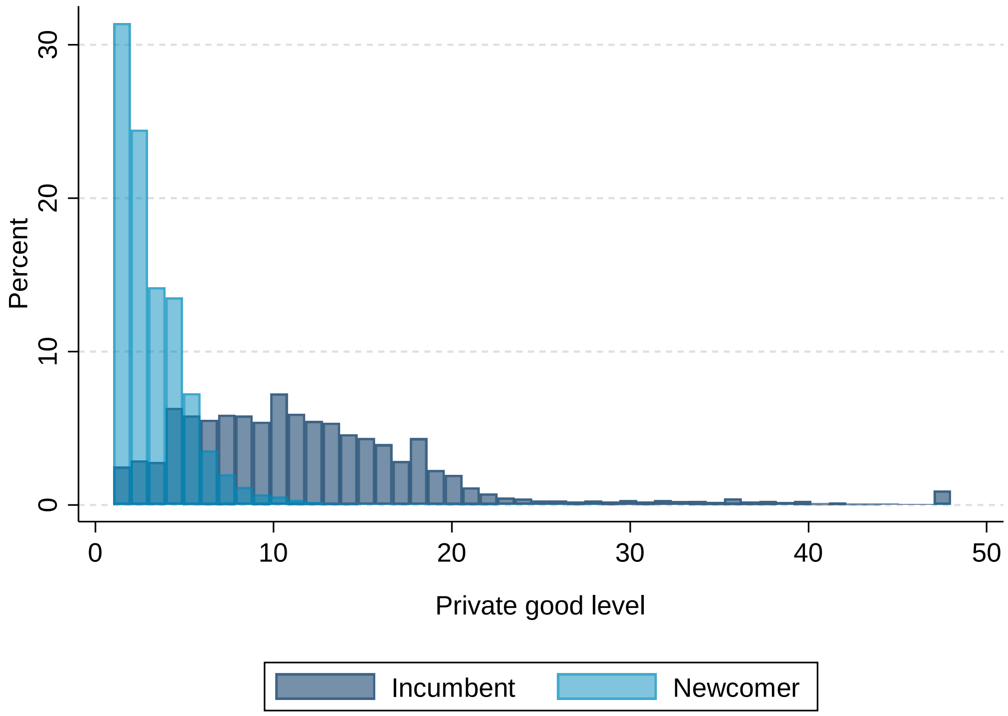

Figure S3. Histogram of private good levels for incumbents and newcomers

Table S17. Individual contribution percentage by group's average contribution

	Model 1	Model 2
Group-average contribution	0.20***	0.21***
	(0.00)	(0.00)
Newcomer	-18.19***	
	(0.14)	
Newcomer ×	-0.25***	
Group-average contribution	(0.00)	
Individual tenure		1.99***
		(0.01)
Individual tenure ×		0.01***
Group-average contribution		(0.00)
Period	0.73***	-0.53***
	(0.01)	(0.01)
Group size	-0.36***	-0.18***
	(0.02)	(0.02)
Intercept	28.44***	28.44***
	(0.04)	(0.04)
N observations	1536892	1536892
R^2 (overall)	0.04	0.06
R^2 (within)	0.04	0.05

Note:* $p < 0.05$, ** $p < 0.01$, *** $p < 0.001$. Linear regression with individual fixed effects to account for repeated measures within individuals. Coefficients are marginal effects with standard errors in parentheses. Statistical significance is calculated using two-sided t-tests. The units of analysis are individuals per period. Model 1 shows that the relationship between individuals' contribution percentage and their groups' average contribution percentage is lower for newcomers (B = -0.25, SE < 0.01, $p < .001$). Model 2 shows that the relationship between individuals' contribution percentage and their groups' average contribution percentage increases with tenure (number of periods spent in the group; B = 0.01, SE < 0.01, $p < .001$). All variables are entered without discretization.

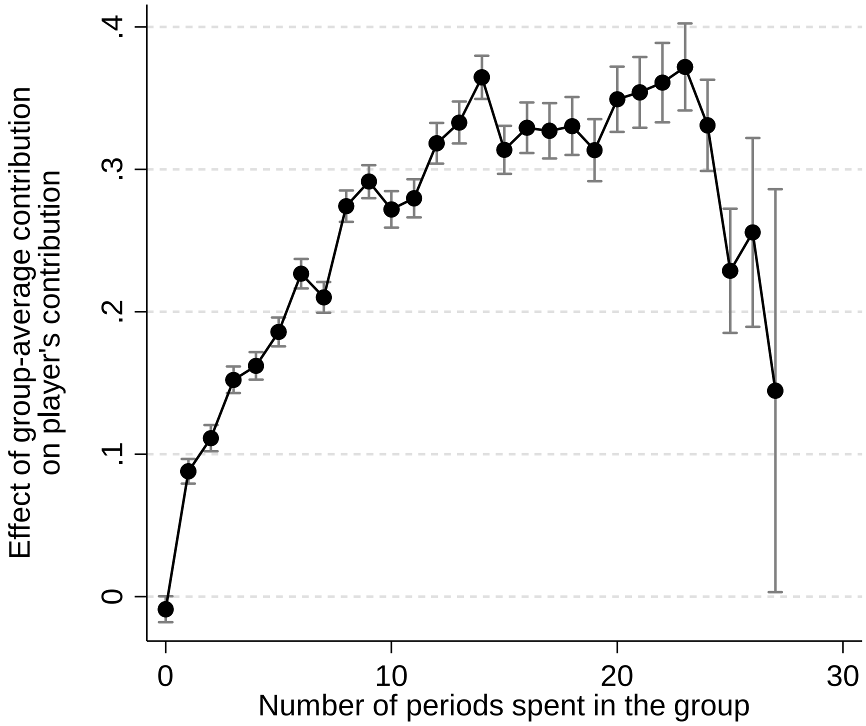

Figure S4. The relationship between players' contribution percentage and their group members' contribution percentage by the players' time spent in the group. Results are derived from a regression model on the individual contribution percentage per period. We account for repeated measures within individuals by estimating individual fixed effects. 95% confidence intervals are provided with vertical capped spikes. Tenure (number of periods spent in the group) is added as a factor variable and interacted with the group's average contribution percentage. We find a clear increase in the relationship between the group's and player's contribution percentage up until a tenure of about 25. After tenure reaches 25, we see a decrease in this relationship, but there are few observations for tenure > 25, leading to large confidence intervals for these final observations. Results include 1536892 contributions decisions made by 134471players. Source data are provided as a Source Data file.

Table S18. Newcomers' contribution percentage by the proportion of incumbents they already know from prior islands

Proportion of incumbents already known	0.74*
	(0.37)
Group size	-0.80***
	(0.03)
Period	-0.68***
	(0.02)
Intercept	23.25***
	(0.48)
N observations	226263
R^2 (overall)	0.01
R^2 (within)	0.01

Note:* $p < 0.05$, ** $p < 0.01$, *** $p < 0.001$. Linear regression with individual fixed effects to account for repeated measures within individuals. Coefficients are marginal effects with standard errors in parentheses. Statistical significance is calculated using two-sided t-tests. The units of analysis are individuals per period. We find a very small effect of newcomers already knowing incumbents from the other islands that they inhabit on their contribution percentage. The difference in the contribution percentage between newcomers who know no incumbents and newcomers who know all incumbents on the new island is only 0.74 percentage points (B = 0.74, SE = 0.37, p = .043). All variables are entered without discretization.

Table S19. Individual contribution percentage for players who belong to only one group

Newcomer	-34.77***
	(0.76)
Group size	3.41***
	(0.18)
Period	-0.19**
	(0.06)
Intercept	7.16**
	(2.19)
N observations	209471
R^2	0.07

Note:* $p < 0.05$, ** $p < 0.01$, *** $p < 0.001$. Linear regression. Coefficients are marginal effects with individual cluster-robust standard errors in parentheses. We have 209471 observations in which players inhabit only one island, i.e., are part of only one group. We find that newcomers have considerably lower contribution percentages than incumbents when selecting players who are part of only one group (B = -34.77, SE = 0.76, $p < .001$). All variables are entered without discretization.

Table S20. Newcomers' contribution percentage by the group-average contributions of their prior group(s)

	Model 1	Model 2
Group-average contribution in most recent prior group	0.02***	
	(0.00)	
Group-average contribution in all prior groups		-0.01*
		(0.01)
Group size	-1.03***	-0.81***
	(0.03)	(0.04)
Period	-0.74***	-0.70***
	(0.02)	(0.02)
Intercept	28.51***	26.09***
	(0.49)	(0.55)
N observations	108256	105351
R^2 (overall)	0.04	0.02
R^2 (within)	0.03	0.02

Note:* $p < 0.05$, ** $p < 0.01$, *** $p < 0.001$. Linear regression with individual fixed effects to account for repeated measures within individuals. Coefficients are marginal effects with standard errors in parentheses. Statistical significance is calculated using two-sided t-tests. The units of analysis are individuals per period. We find very small effects of the group-average contribution in prior groups on the newcomers' contribution in their current group. A one-percentage-point increase in the prior groups' contribution percentage is associated with a 0.02 increase (B = 0.02, SE = < 0.01, p < .001) or 0.01 decrease in the newcomers' contribution percentage (B = -0.01, SE = 0.01, p = .016), depending on whether we take the average contribution percentage of the newcomers' most recent prior group or the average over all prior groups. In both cases, effects are so small as to consider them negligible. All variables are entered without discretization.

Table S21. Individual contribution percentage by newcomer status, with and without controlling for the private good level

	Model 1	Model 2	Model 3
Newcomer	-18.22***	-16.88***	-8.63***
	(0.13)	(0.16)	(0.19)
Period	0.70***	0.93***	0.70***
	(0.01)	(0.02)	(0.02)
Group size	-0.61***	-0.67***	-0.92***
	(0.01)	(0.02)	(0.02)
Private good level			2.93***
			(0.03)
Intercept	27.79***	27.05***	14.94***
	(0.26)	(0.39)	(0.41)
N observations	1572734	898030	898030
R^2 (overall)	0.01	0.01	0.01
R^2 (within)	0.03	0.03	0.04

Note:* $p < 0.05$, ** $p < 0.01$, *** $p < 0.001$. Linear regression with individual fixed effects to account for repeated measures within individuals. Coefficients of independent variables are marginal effects with standard errors in parentheses. Statistical significance is calculated using two-sided t-tests. The units of analysis are individuals per period. Model 1 does not control for private good level; Model 2 also does not control for private good level but includes only observations with private good levels up to 10; Model 3 controls for private good level and includes only observations with private good levels up to 10. Comparing Model 1 and 2, we see that limiting the analysis to observations up to private good levels of 10 does not appreciably change the newcomer-incumbent difference in the contribution percentage. Comparing Model 2 and 3, we see that controlling for private good level roughly halves the newcomer-incumbent difference in contribution percentage. When controlling for private good level, we limit the analysis to private good levels of up to 10 because there are not enough newcomers with private good levels above 10 to reliably compare them with incumbents per private good level (~1%, see also Figure S3). All variables are entered without discretization.

Table S22. Individual contribution percentage by newcomer status and public good level

	Model 1	Model 2
Newcomer	-18.22***	-9.44***
	(0.13)	(0.23)
Period	0.70***	-0.68***
	(0.01)	(0.01)
Group size	-0.61***	-1.46***
	(0.01)	(0.02)
Public good level		2.35***
		(0.02)
Newcomer × Public good level		-0.89***
		(0.03)
Intercept	27.79***	32.50***
	(0.26)	(0.27)
N observations	1572734	1572734
R^2 (overall)	0.01	0.03
R^2 (within)	0.03	0.04

Note:* $p < 0.05$, ** $p < 0.01$, *** $p < 0.001$. Linear regression with individual fixed effects to account for repeated measures within individuals. Coefficients in Model 1 are marginal effects with standard errors in parentheses. Statistical significance is calculated using two-sided t-tests. The units of analysis are individuals per period. The main effect of the newcomer variable in Model 2 is the effect of being a newcomer (instead of incumbent) at the lowest public good level (1). The difference between newcomers and incumbents increases with higher public good levels, as indicated by the negative interaction between the newcomer variable and the public good level (B = -0.89, SE = 0.03, $p < .001$). All variables are entered without discretization.

Supplementary References

1. Asher, S., Casaburi, L., Nikolov, P. & Ye, M. *One step at a time: Do threshold patterns matter in public good provision?* (2009).
2. Ye, M., Zheng, J., Nikolov, P. & Asher, S. One step at a time: Does gradualism build coordination? *Manage. Sci.* **66**, 113–129 (2020).
3. Croson, R. T. A. & Marks, M. B. Step returns in threshold public goods: A meta- and experimental analysis. *Exp. Econ.* **2**, 239–259 (2000).
