# Supplementary material for: Human cooperation in changing groups in a large-scale public goods game
Source: Nat Commun. 2022 Oct 27;13:6399. doi: 10.1038/s41467-022-34160-5 (PMC9613774; doi:10.1038/s41467-022-34160-5)
Supplement: Supplementary file 3 — Description of Additional Supplementary Files [file 41467_2022_34160_MOESM3_ESM.pdf]

Description of Additional Supplementary Files

Supplementary Code: The file ‘syntax-for-creating-figures.do’ is a syntax file using the software Stata. Stata is statistical software for data analysis and can be purchased at https://www.stata.com/. We used version Stata MP 15.1, but the syntax should also work for other versions. The syntax will produce the figures with means/averages presented in the manuscript and supplementary material based on the source data file.
