# Supplementary material for: Human cooperation in changing groups in a large-scale public goods game
Source: Nat Commun. 2022 Oct 27;13:6399. doi: 10.1038/s41467-022-34160-5 (PMC9613774; doi:10.1038/s41467-022-34160-5)
Supplement: Supplementary file 5 — Reporting Summary [file 41467_2022_34160_MOESM5_ESM.pdf]

nature portfolio

Corresponding author(s): Kasper Otten

Last updated by author(s): Jul 1, 2022

Reporting Summary

Nature Portfolio wishes to improve the reproducibility of the work that we publish. This form provides structure for consistency and transparency in reporting. For further information on Nature Portfolio policies, see our Editorial Policies and the Editorial Policy Checklist.

Statistics

For all statistical analyses, confirm that the following items are present in the figure legend, table legend, main text, or Methods section.

n/a	Confirmed	
☐	☒	The exact sample size (n) for each experimental group/condition, given as a discrete number and unit of measurement
☐	☒	A statement on whether measurements were taken from distinct samples or whether the same sample was measured repeatedly
☐	☒	The statistical test(s) used AND whether they are one- or two-sided<br>*Only common tests should be described solely by name; describe more complex techniques in the Methods section.*
☐	☒	A description of all covariates tested
☐	☒	A description of any assumptions or corrections, such as tests of normality and adjustment for multiple comparisons
☐	☒	A full description of the statistical parameters including central tendency (e.g. means) or other basic estimates (e.g. regression coefficient) AND variation (e.g. standard deviation) or associated estimates of uncertainty (e.g. confidence intervals)
☐	☒	For null hypothesis testing, the test statistic (e.g. F, t, r) with confidence intervals, effect sizes, degrees of freedom and P value noted<br>*Give P values as exact values whenever suitable.*
☒	☐	For Bayesian analysis, information on the choice of priors and Markov chain Monte Carlo settings
☒	☐	For hierarchical and complex designs, identification of the appropriate level for tests and full reporting of outcomes
☒	☐	Estimates of effect sizes (e.g. Cohen's d, Pearson's r), indicating how they were calculated

Our web collection on statistics for biologists contains articles on many of the points above.

Software and code

Policy information about availability of computer code

Data collection	We did not use software for data collection in this study.
Data analysis	The data were analysed using Stata MP 15.1.

For manuscripts utilizing custom algorithms or software that are central to the research but not yet described in published literature, software must be made available to editors and reviewers. We strongly encourage code deposition in a community repository (e.g. GitHub). See the Nature Portfolio guidelines for submitting code & software for further information.

Data

Policy information about availability of data

All manuscripts must include a data availability statement. This statement should provide the following information, where applicable:

- Accession codes, unique identifiers, or web links for publicly available datasets
- A description of any restrictions on data availability
- For clinical datasets or third party data, please ensure that the statement adheres to our policy

The raw data is property of Gameforge, the producer of the game. While Gameforge allowed the authors to use the raw data for academic purposes, it did not allow sharing it in any way. We provide an aggregated dataset at https://osf.io/3wye9/.

Field-specific reporting

Please select the one below that is the best fit for your research. If you are not sure, read the appropriate sections before making your selection.

☐ Life sciences ☒ Behavioural & social sciences ☐ Ecological, evolutionary & environmental sciences

For a reference copy of the document with all sections, see nature.com/documents/nr-reporting-summary-flat.pdf

Behavioural & social sciences study design

All studies must disclose on these points even when the disclosure is negative.

Study description	We study longitudinal game data from the multiplayer online game Ikariam. Ikariam is a game in which public good problems are deliberately built in by the designers and central to success. The data are quantitative and span roughly 10 months.
Research sample	The research sample consists of about 135 thousand players of the Ikariam game. Results from a survey suggest that about 80 percent of the players are male, with an average age of 31 years. Compared to typical research using public good games, our sample is broader and more heterogeneous. The inclusion of players from Germany, the United Kingdom, France, Greece, and Turkey means our sample goes slightly beyond typical WEIRD samples (Western, Educated, Industrialized, Rich, and Democratic). The average age of around 31 years suggests that our sample more closely reflects the global median age than most other public good game studies which predominantly recruit younger university undergraduates. Whereas social dilemma studies are typically somewhat overrepresented by women, Ikariam is largely overrepresented by men as is common for computer games. We do not have access to data on the income or education of Ikariam players, so cannot establish representativeness in these aspects. Altogether, our sample presents an improvement in terms of representativeness in some areas (e.g., global coverage and age), but still has limited representativeness in other areas (e.g., sex). The research sample is provided by Gameforge, the producer of the Ikariam game.
Sampling strategy	Data cover the population of five servers in Ikariam. We have data from about 1.5 million contribution decisions made by 135 thousand players in 11.3 thousand groups. This sample size is extensive compared to other studies on public good games, which typically have fewer than a thousand participants.
Data collection	Data were collected through snapshots of observed behavior in the virtual world of Ikariam (i.e., unobtrusive observation of contribution decisions by Ikariam players). We have data from five servers, each from a different country: Germany, United Kingdom, France, Greece, and Turkey. Data collection is identical and synchronous for each country. The data is structured in 28 biweekly snapshots between April 2013 and February 2014 (the snapshots are biweekly on average; they start out weekly in April, get biweekly in August, and still later it is a four-week interval). The first snapshot coincides with the start of new game servers, so we begin our observation at the actual beginning of a game. Players of the game provide consent for (third-party) analyses of their non-personal data when signing up for the game. We did not have access to any personal data. Data were collected by Gameforge, the producer of the game. Gameforge was unaware of the research questions/predictions.
Timing	Between April 2013 and February 2014.
Data exclusions	Snapshots after the 28th snapshot are excluded because no newcomers entered in these snapshots and a prerequisite for our study is newcomer entry.
Non-participation	Data cover the population of five servers in Ikariam, so there is no non-participation.
Randomization	This is an observational study, so there was no randomization.

Reporting for specific materials, systems and methods

We require information from authors about some types of materials, experimental systems and methods used in many studies. Here, indicate whether each material, system or method listed is relevant to your study. If you are not sure if a list item applies to your research, read the appropriate section before selecting a response.

Materials & experimental systems

n/a	Involved in the study	
☒	☐	Antibodies
☒	☐	Eukaryotic cell lines
☒	☐	Palaeontology and archaeology
☒	☐	Animals and other organisms
☐	☒	Human research participants
☒	☐	Clinical data
☒	☐	Dual use research of concern

Methods

n/a	Involved in the study	
☒	☐	ChIP-seq
☒	☐	Flow cytometry
☒	☐	MRI-based neuroimaging

Human research participants

Policy information about studies involving human research participants

Population characteristics	See above.
Recruitment	See above.
Ethics oversight	We obtained ethical approval from the Faculty Ethics Review Board of the Faculty of Social and Behavioural Sciences of Utrecht University.

Note that full information on the approval of the study protocol must also be provided in the manuscript.
